# Image dataset of urine test results on petri dishes for deep learning classification

**DOI:** 10.1016/j.dib.2023.109034

**Published:** 2023-03-02

**Authors:** Gabriel Rodrigues da Silva, Igor Batista Rosmaninho, Eduardo Zancul, Vanessa Rita de Oliveira, Gabriela Rodrigues Francisco, Nathamy Fernanda dos Santos, Karin de Mello Macêdo, Amauri José da Silva, Érika Knabben de Lima, Mara Elisa Borsato Lemo, Alessandra Maldonado, Maria Emilia G. Moura, Flávia Helena da Silva, Gustavo Stuani Guimarães

**Affiliations:** a University of São Paulo, School of Engineering, Av. Prof. Luciano Gualberto, 1380, Cidade Universitária, São Paulo-SP, 05508-010, Brazil; b Grupo Fleury – Clinical Analysis, Av. Morumbi, 8860, São Paulo-SP, Brazil

**Keywords:** Image Classification, Computational Vision, Urine Test Classification, Petri Dish

## Abstract

Recent advancements in image analysis and interpretation technologies using computer vision techniques have shown potential for novel applications in clinical microbiology laboratories to support task automation aiming for faster and more reliable diagnostics. Deep learning models can be a valuable tool in the screening process, helping technicians spend less time classifying no-growth results and quickly separating the categories of tests that deserve further analysis. In this context, creating datasets with correctly classified images is fundamental for developing and improving such models. Therefore, a dataset of urine test Petri dishes images was collected following a standardized process, with controlled conditions of positioning and lighting. Image acquisition was conducted by applying a hardware chamber equipped with a led lightning source and a smartphone camera with 12 MP resolution. A software application was developed to support image classification and handling. Experienced microbiologists classified the images according to the positive, negative, and uncertain test results. The resulting dataset contains a total of 1500 images and can support the development of deep learning algorithms to classify urine exams according to their microbial growth.

**Specifications Table**

**Table:** 

Subject	Computer vision and pattern recognition.
Specific subject area	Image classification, urine test results classification.
Type of data	Image.
How the data were acquired	Images were acquired with a Samsung Galaxy Note 9 mobile phone with a 12 MP AF sensor (sensor rate of 4:3, sensor size of 1/3.4” and pixel size of 1.0µm). The cell phone was coupled in a hardware acrylic cubic box of 306 mm wide, height, and depth. The collected images were saved in a cloud database using a mobile application developed in React Native and Firebase.
Data format	The data are in jpeg format.
Description of data collection	The mobile phone was fitted into an acrylic chamber so that the Petri dishes were always placed centrally, at a fixed distance from the camera, and with standard artificial lighting using a 26cm diameter LED ring light (the chamber CAD file is provided in the Mendeley repository). A square cut has been made in the images before saving them to keep the Petri dish centered and eliminate the empty spaces of the rectangular sensor. A microbiologist classified the collected images. Additional information was included as metadata to justify the classification according to a standard.
Data source location	Institution: Fleury S.A. City/Town/Region: São Paulo, São Paulo state Country: Brazil Latitude -23.63765711436827 and longitude -46.64565897448785
Data accessibility	Repository name: Image Dataset of Clinical Urine Test Results on Petri Dishes Data identification number: 10.17632/xrbtd74pfj.3 Direct URL to data: http://dx.doi.org/10.17632/xrbtd74pfj.3[1]

## Value of the Data

• The dataset provides labeled images of urine exam results, addressing this field's lack of image datasets.
• The data can be applied by researchers and practitioners aiming to develop novel solutions to classify microbiology analysis results based on image recognition of bacterial colonies’ growth on Petri dishes.
• This dataset can contribute to the creation of novel deep learning algorithms that enable the classification of clinical urine cultures in agar plates.
• The dataset can be applied to evaluate the performance of alternative agar plates’ classification algorithms based on image recognition.
• The procedures, hardware, and software developed to collect the dataset can be adapted to similar contexts to construct Petri dishes images datasets.

## 1 Data Description

High-quality digital images of the bacterial cultures on solid agar plates are triggering a new digital revolution in the microbiology field [2]. Novel applications using deep learning algorithms can support task automation in clinical laboratories, not only helping technicians spend less time classifying no-growth results and focusing on positive or complex specimens that deserve further analysis [3,4], as well as leading to more efficient and precise diagnostics [2,5]. Examples of the use of computer vision combined with artificial intelligence techniques in laboratory practice include the detection of hemolyzed samples and parasites in blood tests [6,7].

However, medical image analysis usually requires sets of supervised data, which are often difficult or expensive to acquire [8]. To face the lack of classified images [8], a dataset is created by collecting and classifying photos of urinalysis tests in Petri dishes. The files are labeled according to the dataset structure, consisting of three subdirectories: Positive, Negative, and Uncertain. Each subdirectory contains image files in jpeg format and annotations in an XLSX file about the respective result of the exams. The distribution of the data in each category is represented in Table 1.

**Table 1:** Exams results and respective quantity of images in the dataset.

Exam result	Number of images
Positive	498
Negative	500
Uncertain	502

The tests’ results were based on uroculture, as urines were seeded in plates of chromogenic medium (CPS elite - Biomerieux), in a quantitative way. The plates were initially incubated for 24h at 35°C for the first screening, and those that remained negative were incubated for another 24h, totaling 48h of incubation.

**Fig. 1:**
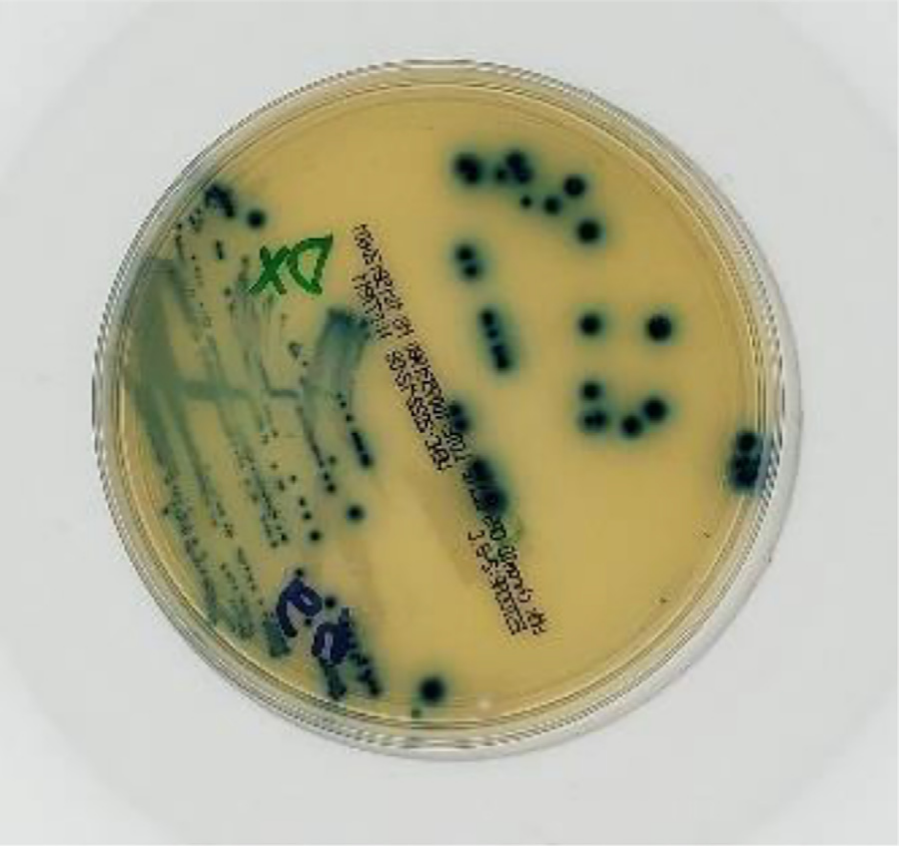
Positive sample image.

A specific description of each test result is provided below. For screening, cultures with growth of the same colonies greater than or equal to 2 Colony-Forming Units (CFU) were considered Positive (example in Fig. 1).

**Fig. 2:**
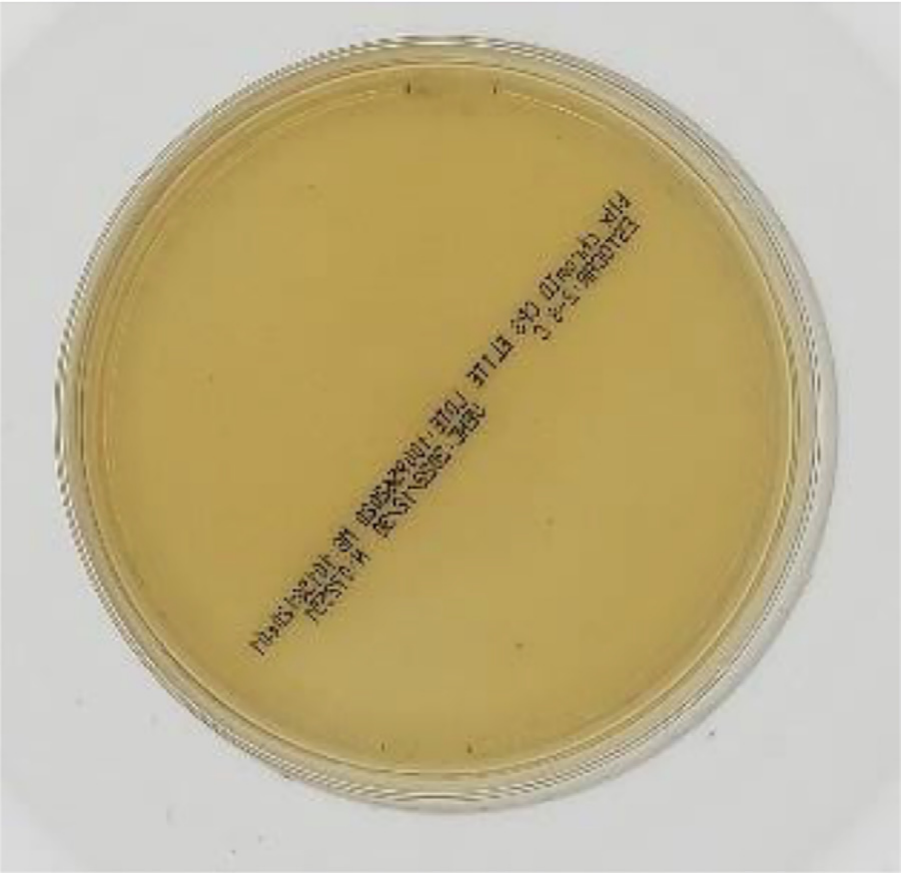
Negative sample image.

Cultures without growth were considered Negative (example in Fig. 2).

**Fig. 3:**
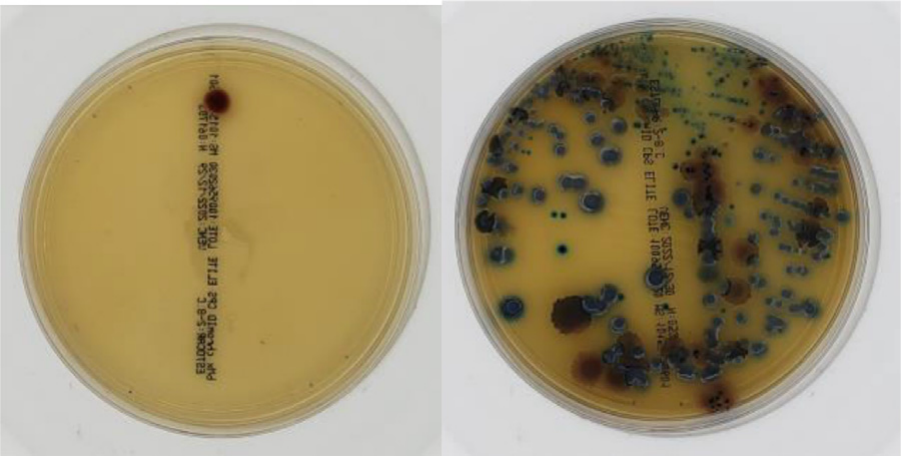
Uncertain sample images with growths of 1 CFU (left) and mixed colonies (right).

Cultures with growth of 1 CFU or growth of mixed colonies were considered Uncertain (example in Fig. 3).

**Fig. 4:**
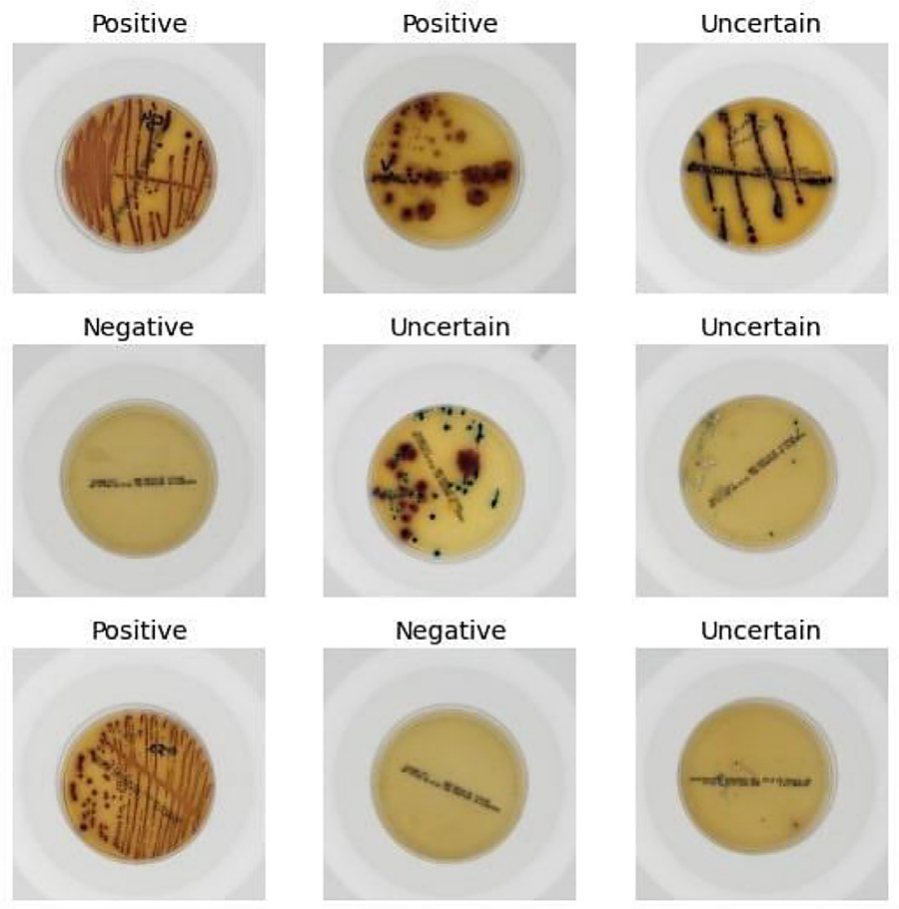
Sample images.

In total, the dataset has 1500 images with 3024 × 3024 pixel resolution each, which is the original camera output resolution considering a central square cut to convert the sensor's ratio from 4:3 to 1:1. Sample images are shown in Fig. 4.

**Fig. 5:**
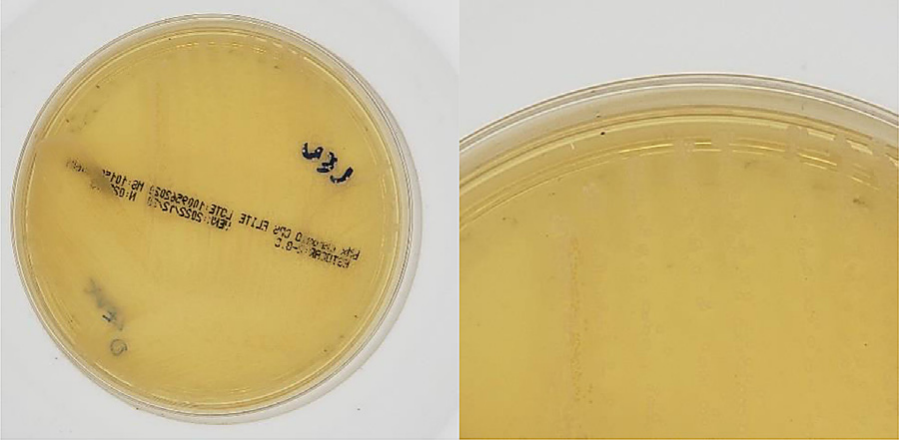
Positive test with colorless microbial colonies.

File sizes range from 600 KB to 1 MB. Although larger files imply greater storage and processing capacity, it was decided to maintain the original image resolution since there are positive results with colorless microbial colonies (Fig. 5). In these situations, low image resolutions may lead to incorrect ratings of these tests as false negatives. This is a consequence of the high variability in bacterial growing patterns that can make a relatively trivial task for microbiologists a very complex task for a machine [2].

The annotations are in XLSX format and bring additional information about each image. The importance of this field is mainly related to bringing more knowledge about the exams, allowing further analyses, such as in the identification of polymicrobial growths. Table 2 provides a summary of the classification parameters and respective labels.

**Table 2:** Classification parameters.

Label	Result	Annotation
A	Positive	2 a 99.000 CFU/mL
B	Positive	> 100.000 CFU/mL
C	Negative	No growth
D	Uncertain	1 CFU/mL
E	Uncertain	Polymicrobial growth

Every image in the dataset was labeled according to the classification parameters, as described in Table 2.

## 2 Experimental Design, Materials and Methods

A specific image acquisition system was developed to support data collection following a standardized process. The system is composed of a tailored hardware chamber integrated with a smartphone providing the camera and a software application.

The chamber consists of a white acrylic cubic box made to set a standard environment for the photos, with appropriate conditions of positioning and lighting. The chamber has a drawer with a positioning swell so that the Petri dishes can be easily positioned and removed. White acrylic 3mm thick sheets were used for chamber manufacturing. The light source is a 26 cm outer diameter ring light with 3800 lumens. The hardware was designed to reduce the interference of ambient light, which could generate different lighting conditions for each image (e.g., images with parts of the plate with the absence of light or the presence of shadows) [9,10]. The CAD file of the chamber is also available in the Mendeley data repository.

**Fig. 6:**
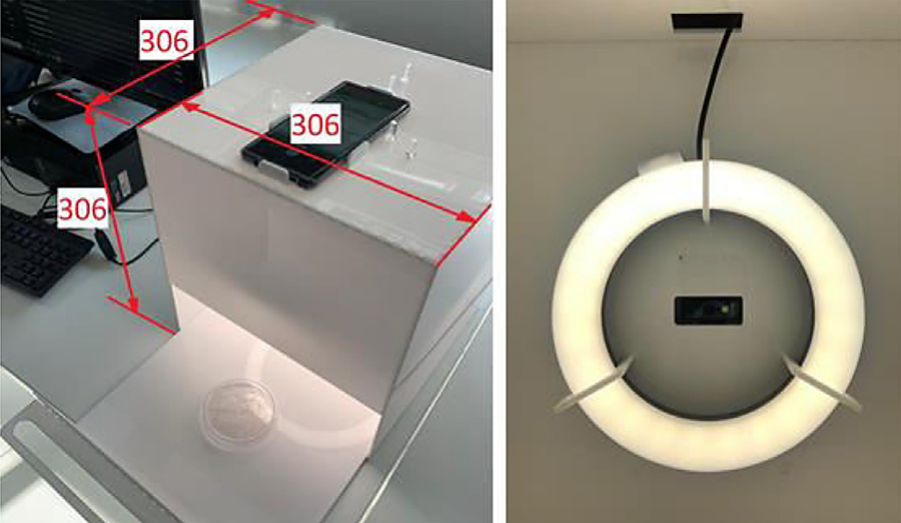
Arrangement of the system with its dimensions and ring light position.

The camera used is embedded in a smartphone (Samsung Galaxy Note 9). The camera has 12 MP resolution (AF sensor, sensor rate of 4:3, sensor size of 1/3.4” and pixel size of 1.0µm). The high-resolution cameras embedded in modern mobile phones can be used for image collection for computer vision solutions. A wide range of possibilities of its use for sensing systems, which includes the acquisition of Petri dishes’ photos, is presented by Grossi [9]. In order to avoid non-uniform camera characteristics, the same smartphone model was used to capture all the images. Fig. 6 presents the system layout and main dimensions.

**Fig. 7:**
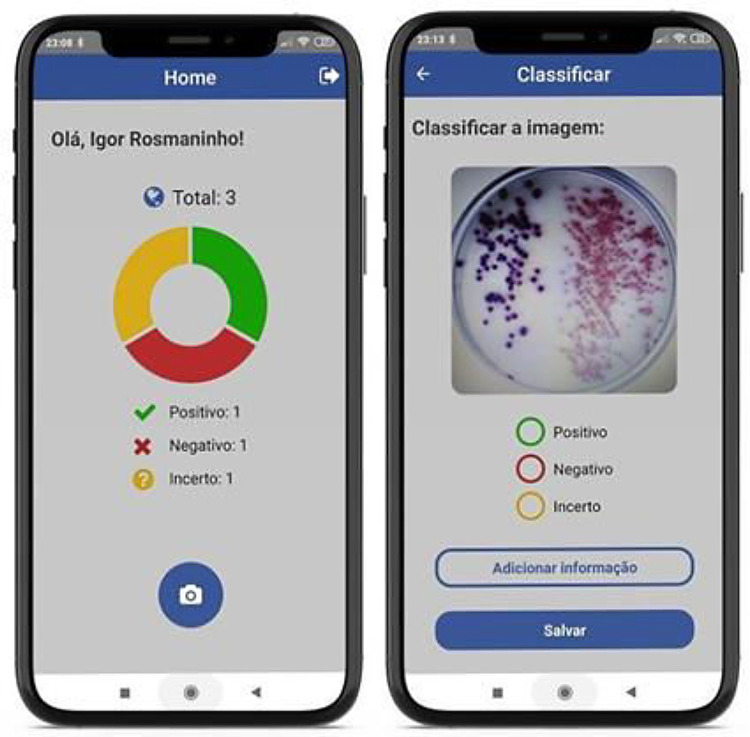
Screenshots of the image acquisition and classification mobile application.

A mobile software application was developed to support image acquisition. The mobile application was developed using React Native as front-end technology and Firebase as back-end-as-a-service. The purpose of the application is to take pictures of the Petri dishes, provide a manual classification function for the user to define the state of the culture among the available labels (Positive, Negative, and Uncertain), and upload the images into the database. The app also allows the inclusion of complementary data for each image in a free text field. Image metadata, including date, time, user ID, is also stored. Moreover, the app provides the number of images that have already been acquired. Fig. 7 presents the classification screen of the mobile software application.

To obtain the final result of this dataset, no filtering or other type of pre-processing was performed.

Beyond the generated dataset, the image acquisition methods employed in this research may serve as a reference for other future works.

## Ethics Statements

The dataset is a result of secondary research using biospecimens not collected specifically for this study. The samples are anonymized and do not contain any identifiable private information. Ethics Committee approval 57858722.0.0000.5474 may/2022.

## CRediT Author Statement

**Gabriel Rodrigues da Silva:** Writing – Original Draft, Software; **Igor Batista Rosmaninho:** Writing – Original Draft, Software; **Eduardo Zancul:** Conceptualization, Supervision, Writing – review & editing, Project administration; **Vanessa Rita de Oliveira:** Investigation, Resources; **Gabriela Rodrigues Francisco:** Investigation, Resources; **Nathamy Fernanda dos Santos:** Investigation, Resources; **Karin de Mello Macêdo:** Investigation; **Amauri José da Silva:** Investigation; **Erika Knabben de Lima:** Investigation; **Mara Elisa Borsato Lemo:** Supervision; **Alessandra Maldonado:** Supervision; **Maria Emilia G Moura:** Supervision; **Flávia Helena da Silva:** Conceptualization, Supervision, Writing – review & editing; **Gustavo Stuani Guimarães:** Supervision.

## Declaration of Competing Interest

The authors declare that they have no known competing financial interests or personal relationships that could have appeared to influence the work reported in this paper.

## Data Availability

Image Dataset of Clinical Urine Test Results on Petri Dishes (Original data) (Mendeley Data).
